# Magnitude of Pulse Pressure Variation is Associated with Qp:Qs Imbalance during Pediatric Cardiac Surgery: A Two-Center Retrospective Study

**DOI:** 10.31083/j.rcm2408242

**Published:** 2023-08-24

**Authors:** Ding Han, Siyuan Xie, Chuan Ouyang

**Affiliations:** ^1^Department of Anesthesia, Capital Institute of Pediatrics Affiliated Children’s Hospital, 100020 Beijing, China; ^2^Anesthesia Center, Capital Medical University Affiliated Beijing Anzhen Hospital, 100029 Beijing, China

**Keywords:** pulse pressure variation, children, cardiac surgery, pressure recording analytical method

## Abstract

**Background:**

Pulse pressure variation (PPV) is based on heart-lung interaction and its association with the imbalance between pulmonary and systemic blood flow (Qp:Qs) has been understudied. We hypothesized that (1) baseline PPV (after induction of anesthesia) is different in a mixed congenital heart disease population with different Qp:Qs, (2) baseline PPV is different between a pooled group with high Qp:Qs and one with low Qp:Qs, and (3) a systemic-pulmonary shunt procedure results in reduced PPV compared to baseline.

**Methods:**

We retrospectively reviewed the medical charts of children who presented to the operating room for cardiac surgery between 2010 and 2018. General patient characteristics, PPV, and other hemodynamic parameters following the induction of general anesthesia were retrieved. Patients were grouped according to the type of congenital heart disease, and whether the Qp:Qs ratio was higher or lower than 1. We also identified patients who received a systemic-pulmonary shunt in order to evaluate changes in PPV.

**Results:**

A total of 1253 patients were included in the study. Baseline PPV differed significantly according to the type of congenital heart disease, with atrial septal defect showing the lowest PPV (9.5 ± 5.6%) and tricuspid valve malformation the highest (21.8 ± 14.1%). The high Qp:Qs group (n = 932) had significantly lower PPV compared to the low Qp:Qs group (n = 321; 11.8 ± 5.7% vs. 14.9 ± 7.9%, respectively; *p* < 0.001). PPV decreased significantly following systemic-pulmonary shunt.

**Conclusions:**

PPV was associated with Qp:Qs imbalance in children undergoing general anesthesia for cardiac surgery. A lower PPV was associated with increased Qp:Qs. Clinicians should take this into account when using PPV to evaluate volume status and when conducting clinical trials in a mixed population of patients with congenital heart disease.

## 1. Introduction

Despite advances in surgical technique, anesthesia and cardiopulmonary bypass management, the perioperative mortality rate for pediatric cardiac surgery remains relatively high, with low cardiac output being a major reason [1, 2]. Meticulous fluid administration is a challenging task in the treatment of critically ill children with low cardiac output. A young age and reduced cardiac function make it difficult to manage perioperative fluid in children with congenital heart disease (CHD). While hypovolemia reduces cardiac output, fluid overload is associated with increased mortality and morbidity in children undergoing cardiac surgery [3]. Many authors recommend the use of dynamic preload parameters derived from arterial waveforms to evaluate fluid status in critically ill patients [4, 5, 6]. These include pulse pressure variation (PPV), stroke volume variation and systolic blood pressure variation, of which PPV is easily accessible, operator-independent, and convenient for continuous monitoring in clinical settings.

PPV is generated mainly because the cyclical squeeze of mechanical ventilation on intrathoracic great vessels and pulmonary vasculatures reduces right ventricle preload, followed several heartbeats later by a decrease in left ventricle preload [7]. During hypovolemia, the preload of the left heart swings periodically and significantly, with the heart working on the steep portion of the Frank-Starling curve. This results in large cyclic alternations in stroke volume, blood pressures, and hence pulse pressure. In this scenario, volume replacement would augment the stroke volume and make the heart work in the flat portion of the Frank-Starling curve, resulting in a small PPV. PPV could therefore be used to predict a fluid response (FR). PPV is based on the heart-lung interaction, and the cyclic change of preload induced by mechanical ventilation originates from the right heart system (intrathoracic great vessels-right atrium-right ventricle-pulmonary-left atrium-left ventricle-large arteries) rather than the left heart system. It therefore seems reasonable to assume that PPV reflects the preload of pulmonary circulation more than that of systemic circulation.

Although PPV is usually used in patients with intact cardiac anatomy, its simple and practical features have encouraged clinicians to deploy PPV in children with CHD [8, 9, 10]. Several previous studies have shown that PPV is useful for the prediction of FR pre- and post-surgical modification in this population [11, 12]. However, the designs of previous studies were limited by the mixed CHD populations and the lack of analysis regarding the effect of circulation imbalance on FR predictivity, thereby decreasing the predictive power of PPV. CHD is a composite term used for a wide spectrum of structural heart diseases and great vessels. It is characterized by an imbalance between pulmonary and systemic blood flow (Qp:Qs), which varies substantially among different types of CHD. The circulation imbalance consists of either higher Qp:Qs or lower Qp:Qs. In the context of preserved cardiac function, hypovolemia results in low stroke volume and high PPV. Intuitively, children with high Qp:Qs should present systemic hypovolemia and hence elevated PPV values compared to children with low Qp:Qs, all other factors being equal. However, as mentioned previously, PPV reflects pulmonary circulation preload more than it reflects systemic circulation preload, therefore the converse of this intuition should be true.

The aim of this study was therefore to investigate the relationship between Qp:Qs imbalance and baseline PPV (after induction of anesthesia) in pediatric patients undergoing general anesthesia for cardiac surgery. Assuming that baseline PPV is associated with Qp:Qs imbalance, we hypothesized that (1) baseline PPV (after induction of anesthesia) was different among different types of CHD, (2) baseline PPV between pooled groups with high Qp:Qs or low Qp:Qs was different, and (3) the establishment of a systemic-pulmonary shunt would increase Qp:Qs dramatically, resulting in a lower PPV.

## 2. Material and Methods

### 2.1 Study Design

We performed a two-center, retrospective observational study of children presenting to the operation room for cardiac surgery between January 2010 to October 2018 at the Children’s Hospital affiliated to Capital Institute of Pediatrics and Beijing Anzhen Hospital, both located in Beijing, China.

### 2.2 Data Sources

Hemodynamic data derived from pressure recording analytical method (PRAM) were retrieved from a dataset containing electronic case report forms which had been collected previously for research purposes. Five graduate students participated in this study as observers and data collectors. PRAM data were merged with the clinical database according to the patient identification number. Patient characteristics collected from the clinical database included age, body weight, body surface area, and major diagnosis of cardiac disease.

### 2.3 Inclusion Criteria

Medical charts were retrieved for children aged <8 years old who underwent cardiac surgery. The inclusion criteria were a tidal volume of 10 mL/kg in routine clinical practice, no use of positive end expiratory pressure, left ventricular ejection fraction >55% estimated during preoperative echocardiogram, and advanced hemodynamic monitoring by MostCare® (Vygon, Vytech, Padova, Italy, powered by PRAM) [13], in addition to standard of care invasive blood pressure monitoring.

### 2.4 Exclusion Criteria

Exclusion criteria were lack of Qp:Qs imbalance, American Society of Anesthesiologists physical classification greater than or equal to IV, prolonged arterial cannulation time (duration from insertion of tracheal tube to artery cannulation >10 min), emergency surgery, and missing data.

### 2.5 Exposure and Grouping

Exposure was the specific type of CHD.

For pooled analysis, the type of CHD was divided into two groups: high Qp:Qs (Qp:Qs ratio >1) and low Qp:Qs (Qp:Qs ratio <1). The high Qp:Qs cardiovascular anomalies included ventricular septal defect, atrial septal defect, total anomalous pulmonary venous connection, patent ductus arteriosus, double outlet of right ventricle, ventricular septal defect combined with atrial septal defect, endocardial cushion defect, partial anomalous pulmonary venous connection, and transposition of the great arteries. The low Qp:Qs cardiovascular anomalies included tetralogy of Fallot, pulmonary atresia, pulmonary stenosis, tricuspid valve malformation (Ebstein anomaly), and other type of CHD (double outlet of right ventricle and transposition of the great arteries) combined with pulmonary stenosis.

For analysis of before-after changes in PPV following establishment of a systemic-pulmonary shunt, all patients in the sub-cohort received the procedure.

### 2.6 Data Acquisition

PRAM data included systolic pressure, diastolic pressure, heart rate, stroke volume index, cardiac index, systemic vascular resistance index, maximal slope of systolic upstroke ${\mathrm{(dp/dt}}_{\text{max}}$), and PPV. The following formula was applied to obtain PPV by PRAM during the most recent 15 s of monitoring period:

$$\mathrm{PPV}\left(\%\right)=\frac{\left(\text{ Maximum Pulse Pressure - Minimum Pulse Pressure }\right)}{\left(\text{ Maximum Pulse Pressure + Minimum Pulse Pressure }\right)/2}\times 100$$

In our previous study procedure, data provided by PRAM was exported to Excel file format for offline analysis. Reasonable data were adopted and documented in a paper clinical record form. The Excel file contained predefined markers indicating the time of intraoperative events, such as artery cannulation, tracheal intubation, surgical incision, etc.

The acquisition time for PRAM data corresponded to the time after arterial cannulation, which was routinely performed after the induction of anesthesia. For patients who received a systemic-pulmonary shunt, data was acquired at the following time points: after the induction of anesthesia, one minute after shunt establishment, 10 minutes after shunt establishment, and at the end of surgery.

### 2.7 Endpoints

The primary endpoint was baseline PPV, while the secondary endpoints were other hemodynamic parameters.

### 2.8 Statistical Analysis

Normally distributed continuous variables were presented as the mean ± SD and compared by independent *t*-test or one-way analysis of variance (ANOVA), with Bonferroni correction for multiple comparisons as appropriate. The mean difference and 95% confidence interval (95% CI) were also reported. Non-normally distributed continuous variables were presented as the median with 25th and 75th percentiles, and compared using the Mann–Whitney test or Kruskal-Wallis as appropriate. Categorical variables were presented as a frequency and compared by Chi-square tests. All statistical tests were two-sided, and statistical significance was defined as a *p* value < 0.05. SPSS 22.0 (IBM Corporation, Armonk, NY, USA) was used for all statistical analyses.

An exploratory before-after analysis was performed in a small subgroup that received the systemic-pulmonary shunt procedure. Paired *t*-tests with Bonferroni correction for multiple comparisons were used to investigate intra-group differences.

Pearson’s correlations between PPV and other variables were also explored. A significant correlation was defined as *p* < 0.05 and |r| >0.3.

## 3. Results

### 3.1 Baseline Characteristics

A total of 1291 children fulfilled the inclusion criteria. After the exclusion of 38 ineligible patients, 1253 patients remained in the study. The reasons for exclusion included an absence of Qp:Qs imbalance (n = 10), prolonged arterial cannulation time (n = 14), and missing data (n = 14).

### 3.2 Comparison of PPV between Different Types of CHD

**Fig. 1.**
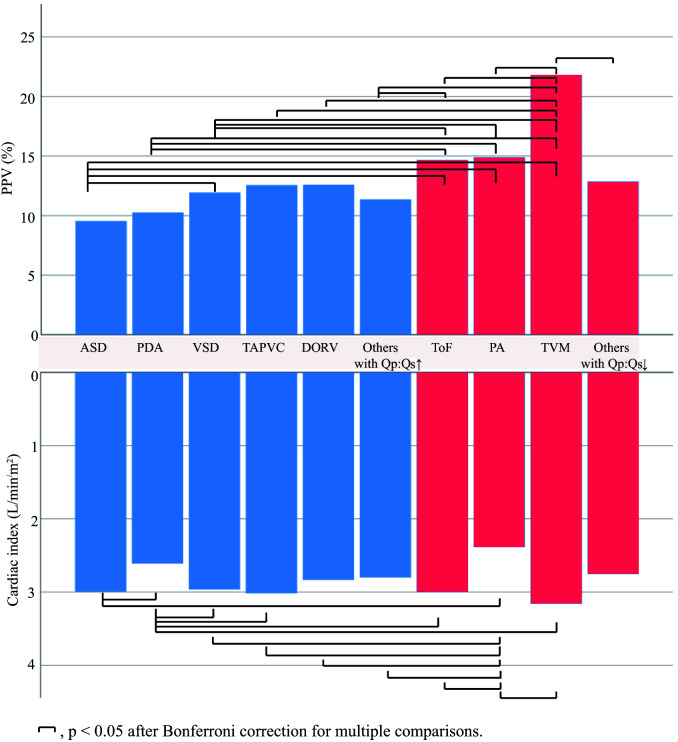
**Baseline mean PPV value according to the type of CHD**. ASD, atrial septal defect; PDA, patent ductus arteriosus; VSD, ventricular septal defect; TAPVC, total anomalous pulmonary venous connection; DORV, double outlet of right ventricle; ToF, tetralogy of Fallot; PA, pulmonary atresia; TVM, tricuspid valve malformation; PPV, pulse pressure variation; CHD, congenital heart disease. Blue color indicates high Qp:Qs and red color indicates low Qp:Qs.

The baseline characteristics of children according to their type of CHD is shown in Table 1. The parameters of age, weight, height, and BSA differed significantly between CHD types (*p* < 0.001 for all). As shown in Table 2 and Fig. 1, PPV was also significantly different between the CHD types (*p* < 0.001).

**Table 1.** **Baseline characteristics for different types of congenital heart disease**.

Type of congenital heart disease	n	Gender (F/M)	Age (years)	Weight (kg)	Height (cm)	BSA ($m^{2}$)
Atrial septal defect	62	34/28	0.95 [0.72, 1.66]	9.1 [7.5, 9.9]	76 [70, 82]	0.42 [0.36, 0.46]
Patent ductus arteriosus	45	32/13	0.54 [0.3, 0.95]	5.9 [4.4, 6.8]	63 [58, 68]	0.32 [0.26, 0.35]
Ventricular septal defect	709	342/367	0.57 [0.38, 1.1]	6.4 [5.5, 8.5]	67 [62, 73]	0.33 [0.30, 0.40]
Total anomalous pulmonary venous connection	50	14/36	0.16 [0.08, 0.27]	4.5 [3.5, 5.4]	56 [52, 60]	0.25 [0.22, 0.28]
Double outlet of right ventricle	26	7/19	0.26 [0.15, 0.52]	4.2 [3.9, 7.2]	60 [57, 64]	0.25 [0.25, 0.33]
Others with increased Qp:Qs	40	20/20	0.44 [0.17, 0.71]	5.2 [4.4, 7.0]	63 [56, 71]	0.29 [0.24, 0.36]
Tetralogy of Fallot	208	86/122	0.87 [0.71, 1.21]	8.7 [7.5, 10.0]	72 [68, 78]	0.40 [0.36, 0.45]
Pulmonary atresia	53	23/30	0.88 [0.25, 2.21]	8.5 [4.2, 11.6]	70 [55, 86]	0.40 [0.24, 0.49]
Tricuspid valve malformation	19	6/13	1.81 [1.46, 5.36]	11.0 [9.2, 16.0]	82 [79, 107]	0.47 [0.44, 0.69]
Others with decreased Qp:Qs	41	14/27	0.84 [0.43, 1.36]	8.5 [6.4, 9.5]	70 [64, 80]	0.39 [0.33, 0.45]
*p* value for intra-group comparison	-	<0.001	<0.001	<0.001	<0.001	<0.001

Data are shown as the median [25th, 75th percentiles]. BSA, body surface area; F/M, female/male.

**Table 2.** **Baseline PPV values among different types of congenital heart disease (%)**.

Type of congenital heart disease	n	PPV (Mean ± SD)	Upper limit of 95% CI of mean value	Lower limit of 95% CI of mean value
Atrial septal defect	62	9.5 ± 5.6	8.1	11.0
Patent ductus arteriosus	45	10.3 ± 4.4	8.9	11.6
Ventricular septal defect	709	11.9 ± 5.6	11.5	12.3
Total anomalous pulmonary venous connection	50	12.6 ± 3.8	11.5	13.6
Double outlet of right ventricle	26	12.6 ± 3.8	11.1	14.1
Others with increased Qp:Qs	40	11.4 ± 7.6	8.9	13.8
Tetralogy of Fallot	208	14.7 ± 8.6	13.5	15.8
Pulmonary atresia	53	14.9 ± 8.4	12.6	17.2
Tricuspid valve malformation	19	21.8 ± 14.1	15.0	28.6
Others with decreased Qp:Qs	41	12.9 ± 6.3	10.9	14.8

PPV, pulse pressure variation.

Post-hoc analysis revealed that children with atrial septal defect had lower PPV than those with ventricular septal defect (*p* = 0.001, difference = 2.4, 95% CI 1.0–3.8), tetralogy of Fallot (*p* < 0.001, difference = 5.1, 95% CI 3.0–7.2), pulmonary atresia (*p* < 0.001, difference = 5.4, 95% CI 3.0–7.7) or tricuspid valve malformation (*p* < 0.001, difference = 12.3, 95% CI 8.9–15.6).

Patients with patent ductus arteriosus had lower PPV than those with tetralogy of Fallot (*p* < 0.001, difference = 4.4, 95% CI 2.3–6.4), pulmonary atresia (*p* < 0.001, difference = 4.6, 95% CI 2.1–7.2) or tricuspid valve malformation (*p* < 0.001, difference = 11.5, 95% CI 8.1–15.0).

Patients with ventricular septal defect had lower PPV than those with tetralogy of Fallot (*p* < 0.001, difference = 2.7, 95% CI 1.7–3.7), pulmonary atresia (*p* < 0.001, difference = 3.0, 95% CI 1.2–4.8) or tricuspid valve malformation (*p* < 0.001, difference = 9.8, 95% CI 6.9–12.8).

Patients with total anomalous pulmonary venous connection had lower PPV than those with tricuspid valve malformation (*p* < 0.001, difference = 9.2, 95% CI 5.8–12.7).

Patients with double outlet of right ventricle had lower PPV than those with tricuspid valve malformation (*p* < 0.001, difference = 9.2, 95% CI 5.4–13.0).

Patients with other types of CHD and with increased Qp:Qs had lower PPV than those with tetralogy of Fallot (*p* = 0.002, difference = 2.8, 95% CI 1.0–4.6) or with tricuspid valve malformation (*p* < 0.001, difference = 10.0, 95% CI 6.6–13.3).

Patients with tetralogy of Fallot, pulmonary atresia, or other types of CHD combined with pulmonary stenosis had lower PPV than those with tricuspid valve malformation (*p* < 0.001, difference = 7.1, 95% CI 4.0–10.1; *p* < 0.001, difference = 6.9, 95% CI 3.5–10.3; *p* < 0.001, difference = 8.9, 95% CI 5.4–12.5, respectively).

The cardiac index was also analyzed because of its close relationship with PPV. Preload plays an important role in determining cardiac output. Within the context of preserved cardiac function, it is likely that a high PPV signifies a hypovolemia status and is accompanied by a relatively low cardiac output/index. The baseline cardiac index differed significantly between different types of CHD (*p* < 0.001), as shown in Fig. 1.

### 3.3 Comparison of the High and Low Qp:Qs Groups 

The baseline characteristics of the two Qp:Qs groups are shown in Table 3. The high Qp:Qs group (n = 932) was comprised of 709 patients with ventricular septal defect, 62 with atrial septal defect, 50 with total anomalous pulmonary venous connection, 45 with patent ductus arteriosus, 26 with double outlet of right ventricle, 10 with ventricular septal defect combined with atrial septal defect, 14 with endocardial cushion defect, 6 with partial anomalous pulmonary venous connection, and 10 with transposition of the great arteries.

**Table 3.** **Comparison of demographic data between high and low Qp:Qs groups**.

Variable	High Qp:Qs group (n = 932)	Low Qp:Qs group (n = 321)	*p* value
Gender (F/M)	446/486	129/192	0.017
Age (years)	0.55 [0.35, 1]	0.89 [0.69, 1.44]	<0.001
Weight (kg)	7.2 ± 3.1	9.3 ± 4.1	<0.001
Height (cm)	69 ± 13	76 ± 16	<0.001
BSA ($m^{2}$)	0.36 ± 0.11	0.43 ± 0.14	<0.001

BSA, body surface area; F/M, female/male.

The low Qp:Qs group (n = 321) consisted of 208 patients with tetralogy of Fallot, 53 with pulmonary atresia, 19 with tricuspid valve malformation, and 41 with other types of cardiovascular anomalies combined with pulmonary stenosis.

As shown in Table 4, the low Qp:Qs group had significantly higher PPV, higher diastolic blood pressure, lower heart rate, higher systemic vascular resistance index, lower cardiac cycle efficiency, higher stroke volume index, and lower ${\mathrm{dp/dt}}_{\text{max}}$ compared with the high Qp:Qs group.

**Table 4.** **Comparison of intraoperative PPV and other hemodynamic parameters between high and low Qp:Qs groups**.

Variable	High Qp:Qs (n = 932)	Low Qp:Qs (n = 321)	Difference (95% CI)	*p* value
Systolic pressure (mmHg)	86 ± 14	87 ± 15	–1.8 (–3.6, 0.1)	0.060
Diastolic pressure (mmHg)	41 ± 8	44 ± 9	–3.6 (–4.7, –2.5)	<0.001
Heart rate (beats/min)	112 ± 17	106 ± 19	6.4 (4.2, 8.6)	<0.001
Systemic vascular resistance index (${\mathrm{dyn\cdot s/cm}}^{5}$${\mathrm{\cdot m}}^{2}$)	1385 ± 335	1557 ± 474	–172 (–219, –124)	<0.001
Cardiac cycle efficiency (unit)	–0.02 ± 0.31	–0.14 ± 0.41	0.12 (0.08, 0.16)	<0.001
Cardiac index (${\mathrm{L/min/m}}^{2}$)	2.9 ± 0.6	2.9 ± 0.8	0.07 (–0.01, 0.14)	0.122
Stroke volume index (${\mathrm{mL/m}}^{2}$)	26.4 ± 7.7	28.0 ± 9.5	–1.6 (–2.6, –0.6)	0.002
${\mathrm{Dp/dt}}_{\text{max}}$ (mmHg/ms)	1.01 ± 0.23	0.94 ± 0.24	0.07 (0.04, 0.10)	<0.001
PPV (%)	11.8 ± 5.7	14.9 ± 7.9	–3.1 (–3.9, –2.2)	<0.001

PPV, pulse pressure variation; ${\mathrm{dp/dt}}_{\text{max}}$, maximal slope of systolic upstroke.

### 3.4 Changes in PPV before and after the Establishment of a Systemic-Pulmonary Shunt 

We identified 46 patients who received a systemic-pulmonary shunt. As shown in Table 5, PPV showed a non-statistically significant decrease one minute after the establishment of a systemic pulmonary shunt (*p* > 0.05), and then decreased significantly 10 minutes later and at the end of surgery (*p* < 0.001 for both).

**Table 5.** **Changes in PPV and in other hemodynamic variables before and after the establishment of a systemic-pulmonary shunt**.

Variable	After induction of anesthesia	One minute after establishment of shunt	Ten minutes after establishment of shunt	End of surgery
Cardiac cycle efficiency (unit)	0.02 ± 0.29	0.02 ± 0.19	0.06 ± 0.35	0.12 ± 0.16
Cardiac index (${\mathrm{L/min/m}}^{2}$)	2.92 ± 0.48	2.63 ± ${\mathrm{0.46}}^{*}$	3.05 ± 0.63	3.04 ± 0.69
Diastolic pressure (mmHg)	46 ± 7	41 ± 8	46 ± 8	46 ± 8
${\mathrm{Dp/dt}}_{\text{max}}$ (mmHg/ms)	1.02 ± 0.25	1.18 ± ${\mathrm{0.25}}^{*}$	1.37 ± ${\mathrm{0.24}}^{*}$	1.32 ± ${\mathrm{0.25}}^{*}$
Heart rate (beats/min)	105 ± 26	124 ± ${\mathrm{19}}^{*}$	133 ± ${\mathrm{23}}^{*}$	133 ± ${\mathrm{23}}^{*}$
PPV (%)	13.0 ± 4.4	11.2 ± 3.0	6.5 ± ${\mathrm{3.1}}^{*}$	6.1 ± ${\mathrm{1.6}}^{*}$
Stroke volume index (${\mathrm{mL/m}}^{2}$)	29.4 ± 9.2	21.5 ± ${\mathrm{7.1}}^{*}$	24.4 ± ${\mathrm{11}}^{*}$	24.2 ± ${\mathrm{10.6}}^{*}$
Systemic vascular resistance index (${\mathrm{dyn\cdot s/cm}}^{5}$${\mathrm{\cdot m}}^{2}$)	1498 ± 285	1656 ± 277	1598 ± 256	1640 ± 310
Systolic pressure (mmHg)	94 ± 12	98 ± 12	110 ± ${\mathrm{12}}^{*}$	108 ± ${\mathrm{14}}^{*}$

*, *p* < 0.05 versus baseline (after induction of anesthesia). PPV, pulse pressure variation; ${\mathrm{dp/dt}}_{\text{max}}$, maximal slope of systolic upstroke.

### 3.5 Pearson Correlation between PPV and Other Variables

PPV was not significantly correlated with any of the demographic or hemodynamic variables examined, according to the prior definition of a significant correlation.

## 4. Discussion

There has been increased interest in the use of dynamic preload parameter to predict FR in ventilated and critically ill pediatric cardiac patients. Despite this, the effect of Qp:Qs imbalance on dynamic preload parameter has not been studied. In this study, we evaluated the effect of a wide and heterogeneous spectrum of CHD on PPV values, in line with the current strategy of precision medicine. The present results suggest that baseline PPV is associated with Qp:Qs imbalance in pediatric patients undergoing cardiac surgery.

The induction of anesthesia frequently leads to hemodynamic depression due to volume depletion from preoperative fasting, as well as vasodilation caused by anesthetics coexisting with the inhibition of myocardial contractility. The values for hemodynamic variables post-induction of anesthesia are usually referred to as the baseline. These serve as a reference for evaluating hemodynamic trends during the period before the onset of cardiopulmonary bypass (CPB), and can also be used to stratify the level of critical care. Further, the hemodynamic variables are relatively stable and reliable during the induction of anesthesia period, as there is little interference from surgical operations.

PPV accurately predicts FR only under restricted conditions. Several factors have been reported to affect the magnitude of PPV and sometimes also its performance to predict FR. First, transpulmonary pressure has a large impact on the magnitude of PPV, which increases with increasing inspiratory pressure or transpulmonary pressure [4, 14]. For patients subjected to protective lung ventilation (small tidal volume plus positive end expiratory pressure), a reduction in transpulmonary pressure has been shown to reduce the overall PPV and to decrease the cutoff value for predicting FR [15]. Although data on transpulmonary pressure or a similar metric such as lung compliance was not available in this study, we presumed the high Qp:Qs group had higher transpulmonary pressure than the low Qp:Qs group. This was because the former usually has reduced lung compliance due to high Qp and therefore needs a higher driving pressure to achieve an equal tidal volume. From this it follows the high Qp:Qs group would have an elevated rather than reduced PPV compared to the low Qp:Qs group. Second, cardiac dysfunction is another major factor that influences the FR prediction of preload parameter [16]. Based on PRAM data, ${\mathrm{dp/dt}}_{\text{max}}$ in both the high and low Qp:Qs groups remained within a range considered to be normal (>0.8 mmHg/ms). Hence, it is likely that cardiac function was preserved in both groups in this study.

In the present study, the rationale underlying the biased baseline PPV should be the difference in Qp:Qs. From this, a higher pulmonary pressure frequently coexisting with high Qp:Qs should be associated with a lower PPV. This result is in sharp contrast with previous findings on adult patients showing that transient elevated pulmonary pressure coexisting with right ventricle dysfunction can lead to false-positive (elevated) PPV in predicting FR [17]. The reason for this discrepancy could be that the high Qp:Qs group in the present study population had normal or even above normal right ventricle function due to functional hypertrophy and infant physiology [18]. One could assume that the relatively healthy status and less hemodynamic fluctuations in the high Qp:Qs group were responsible for the lower PPV. However, our results showed that the low Qp:Qs group had a comparable cardiac index, a higher stroke volume index, and a lower heart rate compared with the high Qp:Qs group, thus indicating a similar or even more stable hemodynamic status. Children in the low Qp:Qs group were older and had higher body weight, probably because the timing of surgical intervention was chosen to be at a later age in order to better cope with the surgical stress. With advancing age the stroke volume and blood pressure increase, while the heart rate decreases. Hence, the low Qp:Qs group had higher stroke volume index, higher diastolic blood pressure, and lower heart rate compared to the high Qp:Qs group. The higher systemic vascular resistance index in the low Qp:Qs group develops so as to accommodate the relatively inferior cardiac function, as reflected by the lower ${\mathrm{dp/dt}}_{\text{max}}$ and cardiac cycle efficiency to maintain higher systemic blood pressure. Moreover, the high Qp:Qs group contained patients with total anomalous pulmonary venous connection and patent ductus arteriosus. These are recognized as two of the most critical cardiac diseases and are characterized by hemodynamic instability frequently necessitating emergency surgery at an early stage of life. Hence, we do not favor this explanation for the PPV discrepancy.

The establishment of a systemic-pulmonary shunt offers a good opportunity to test our hypothesis that PPV is associated more with Qp than with Qs. We found that a systemic to pulmonary shunt was associated with a trend for reduced PPV one minute after establishment of the shunt, instead of the anticipated elevation in PPV due to the pulmonary system taking blood from the systemic circulation. This reduction was unlikely to be related to volume expansion. As per our anesthesia protocol, only a small volume was given to avoid a full-loaded heart, thereby facilitating surgical manipulation prior to declamping the shunt. The PPV decreased significantly 10 minutes later, probably due to the start of extensive volume replacement therapy and further increases in Qp.

### 4.1 Clinical Implications

The results of this study may have practical value for when clinicians use PPV to discriminate fluid responders. Previous studies have delineated a wide range of threshold values for predicting FR across a large spectrum of CHD [9, 11, 19]. High Qp:Qs was associated with an overall decrease in PPV. Therefore, if PPV was able to predict FR in this population, the low Qp:Qs group would likely have a higher threshold of PPV for predicting FR compared to the high Qp:Qs group. Clinicians should take this bias into account when performing fluid loading based on parameters involving PPV and when conducting clinical trials with a mixed CHD population.

PPV may offer an alternative for estimating the degree of Qp:Qs imbalance in children with high Qp:Qs. It is well recognized that a low diastolic pressure signifies a large systemic to pulmonary shunt in cases presenting with patent ductus arteriosus. Moreover, diastolic pressure has been employed to assist surgeons in determining the amount of artificial shunt in systemic-pulmonary shunt procedures. Similarly, PPV is promising not only for predicting FR but also as a potential indicator of Qp in children with increased Qp.

### 4.2 Limitations 

This study has several limitations. First, it was retrospective in design and therefore subjected to the same limitations as any retrospective study. The results may be biased due to the failure to account for all confounders, such as the duration of preoperative fasting and other unmeasured factors.

Second, the number of subjects with different types of CHD varied. The study population consisted predominantly of ventricular septal defect for the high Qp:Qs group, and tetralogy of Fallot for the low Qp:Qs group. There was an increased risk of sampling error due to the small sample size for some types of CHD.

Third, children with low Qp:Qs were severe patients at risk of hemodynamic instability, and anesthesiologists therefore tend to perform fluid loading upon induction of general anesthesia. Additionally, vasoactive agents may also have been used as needed. The retrospective nature of this study meant these events were not thoroughly documented. However, in either scenario, PPV would have been higher in the low Qp:Qs group if a fluid loading or vasoactive agent was not used. Hence, the difference in PPV at baseline between the high and low Qp:Qs groups may have been reduced.

Fourth, baseline characteristics between the high and low Qp:Qs groups were not adjusted to match. However, general characteristics are not confounders of PPV magnitude according to our prior knowledge and correlation analyses.

Lastly, this study involved patients with intra- or extra-cardiac shunts, which could affect heart-lung interactions and hence the magnitude of PPV. However, the authors consider that PPV is related to preload status when patients are hemodynamically stable and intra- or extra-cardiac shunts remain in a homeostatic state. Indeed, previous studies have reported that dynamic preload parameter was predictive of FR in this study population [11, 12].

## 5. Conclusions

In children with imbalanced Qp:Qs undergoing general anesthesia for cardiac surgery, the intraoperative baseline PPV differed significantly between those with different types of CHD. Moreover, PPV was significantly lower in children with high Qp:Qs compared to those with low Qp:Qs. Imbalanced Qp:Qs contributes to the difference in baseline PPV, thereby potentially affecting the cutoff values for predicting FR. We envision two general directions for future prospective research. First, the theory outlined in this study could be strengthened by evaluating the association between PPV and pulmonary pressures (including pulmonary artery wedge pressure), as measured by pulmonary catheterization. The second future research area is to determine the optimal cutoff value for PPV (if it exits) to predict FR in different types of CHD.

## Data Availability

The datasets used in the current study are available from the corresponding author upon reasonable request.
